# Exploration of Herbal Medicinal Plants and Formulations Available for the Treatment of PCOS: A review

**DOI:** 10.5935/1518-0557.20250006

**Published:** 2025

**Authors:** Arjoo Babhare, Deweshri Nandurkar, Kishor Danao, Amol Warokar, Ujwala Mahajan

**Affiliations:** 1 Dadasaheb Balpande College of Pharmacy, Nagpur - 440037, Maharashtra, India

**Keywords:** polycystic ovary syndrome, treatment, herbal plants, formulations

## Abstract

Polycystic ovarian syndrome (PCOS) is a heterogeneous endocrine disorder with the underlying consequence of ovarian cysts, anovulation, and endocrine variation affecting women. According to the World Health Organization (WHO) estimation over 116 million women (3.4%) are affected by PCOS worldwide. It is typified by polycystic ovaries, hyperandrogenism, and ovulatory dysfunction. Pharmacological interventions that treat symptoms but may have fewer side effects are frequently used in conventional treatments. As a result, interest in herbal medicinal plants and formulations as additional or alternative PCOS therapies is developing. This review explores the safety and efficacy of various herbal remedies traditionally used in the management of PCOS. Each of the herbs mentioned in this review offers unique benefits. This review gives a comprehensive idea about PCOS, its causes and symptoms. It also explores formulations like capsules, tablets, syrups, and nano drug delivery system medicines for PCOS. According to studies, these herbal medicines can improve insulin resistance, lower testosterone levels and restore menstrual regularity while causing fewer negative effects than conventional therapy. .

## INTRODUCTION

In 1935, Leventhal and Stein characterized the condition that later became known as polycystic ovary syndrome (PCOS) (Trivax & Azziz, 2007). Polycystic Ovarian Syndrome is a prevalent and complex illness that affects women throughout their reproductive years. Approximately 70% of women with this disease experience lower fertility due to ovulation issues. Polycystic Ovarian Syndrome is characterized by the occurrence of cysts on the ovaries, preventing normal ovarian function. This disorder has several underlying symptoms, such as obesity or increased weight, high blood pressure, diabetes, dysfunction of the lipid profile, dandruff on the scalp or oily skin, dark-colored patches on the skin of the neck and under the arms, acne, generally chronic pelvic pain, increased levels of male hormones resulting in thinning hair, male pattern baldness, excessive hair growth on the body and face, and, in the case of women, frequent, irregular bleeding, no or infrequent ovulation; and immature follicles (Dayani Siriwardene *et al.*, 2010).

According to the National Institute of Health Office of Disease Prevention, PCOS is a diverse illness that primarily affects women of childbearing age. There are around 5 million cases, accounting for nearly 7% of adult women. PCOS is the most frequent endocrine condition affecting girls aged 18 to 44 years, accounting for 5-10% of females according to a study (Ndefo *et al.*, 2013). Oxidative stress (OS), has been linked to an increase in inflammation and may be the cause of PCOS (Agarwal *et al.*, 2008). Improper metabolism of estrogen, testosterone, and restricted testosterone production are the main underlying causes associated with PCOS in women (Jitendra & Pravin, 2012).

**Figure 1:**
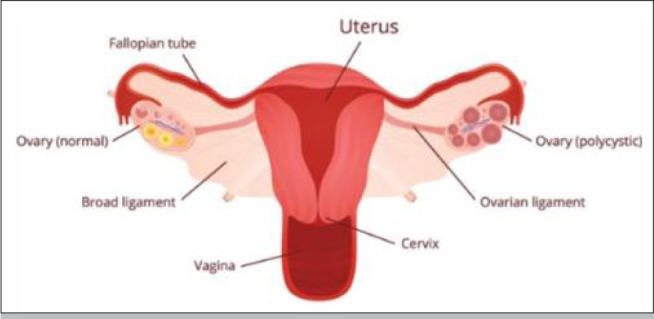
Normal and Polycystic ovary.

Women with PCOS experience anxiety and depression more frequently than people in general. It is widely recognized that mood problems may negatively impact quality of life. This is especially true for young adult women who are worried about fertility, as well as for women of all ages who are concerned about obesity and the clinical signs of excessive androgen (Bharathi *et al.*, 2017; Dumesic *et al.*, 2015). The difference between a normal and polycystic ovary is presented in Figure 1.

### Symptoms (Khanage *et al.*, 2019)

The symptoms of PCOS may include

- Absent periods
- Acne not associated with puberty
- Darkening of the skin
- Excessive hair growth
- Fatigue Fluid retention
- Menstrual pain
- Mood swings
- Ovarian cysts
- Weight gain
- Heavy or prolonged periods
- Infertility/Irregular periods
- Male pattern baldness

### Risk Factors (Begum *et al.*, 2017)

Women are more likely to develop PCOS if she is having the following risk factors. These factors affect and are also associated with secondary complications like type 2 diabetes, high blood pressure, and uterine cancer.

- Obesity
- Family history of Infertility
- Family history of PCOS
- Family history of diabetes
- Fast food diet habits
- Lack of physical exercise

### PCOS Pathophysiology

**Figure 2:**
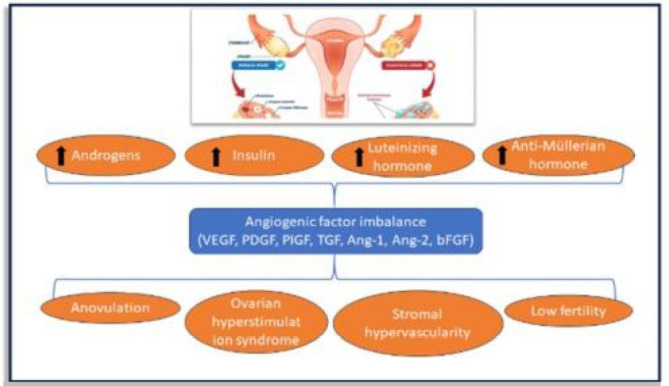
Pathophysiology of PCOS.

Pituitary gonadotropin is essential for reproductive function. It is produced and secreted by the pituitary in response to direct stimulation from the hypothalamus GnRH and integrated feedback processes (Balen, 2004). FSH supplies the first stimulus for follicular growth as well as the stimulation of aromatase enzymes; which convert androgens to estrogens in granulosa cells. Luteinizing Hormone (LH) is most recognized for its ability to increase progesterone secretion during the luteal phase. It also plays a critical function in the follicular phase by stimulating the synthesis of testosterone. Theca cell androgen secretion may rise because a PCOS-afflicted women’s increased LH output. Pulsatile GnRH stimulation is necessary to sustain gonadotropin production; however, prolonged exposure to GnRH leads to desensitization and a decrease in gonadotropin secretion in the pituitary. Over the menstrual cycle, variations in the pulsatility of gonadotropin-releasing hormone (GnRH) affect the ratio of secretion of the two pituitary gonadotropins as explained in Figure 2 (Balen, 2004). In PCOS, excess androgen is linked to a rise in belly fat, which causes hyperinsulinemia and dyslipidemia. To raise the amounts of circulating bioactive testosterone, hyperinsulinemia decreases the amount of hepatic sex hormone-binding globulin (SHBG) (Dumesic *et al.*, 2015). The primary characteristics of PCOS include hypogonadism, elevated amounts of androgens or male sex hormones like testosterone, Acne, male-pattern baldness, and male-pattern hair growth (hirsutism) can result from this (androgenic alopecia) (Kumari *et al.*, 2024) Women with PCOS typically experience irregular or non-existent menstrual periods due to ovulation issues. This makes it harder to become pregnant. During an ultrasound scan, the ovaries may seem swollen and contain many tiny follicles that are sometimes referred to as “cysts.” In actuality, these are immature follicles that have not fully matured (Kumari *et al.*, 2024).

### Associated Clinical Manifestations (Kumari *et al.*, 2024)

**Figure 3:**
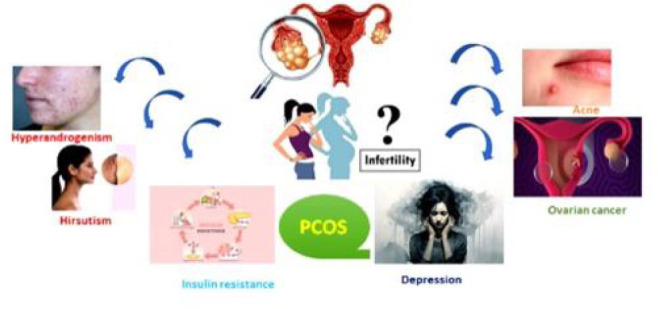
Clinical manifestations with PCOS.

PCOS increases the short-term and long-term risk of endometrial cancer, anxiety, depression, recurrent abortion, and possibly breast cancer, as well as the long-standing risk of obesity, type 2 diabetes, metabolic syndrome, hypertension, dyslipidemia, thyroid, and hyperplasia. These complications’ clinical symptoms include infertility, hirsutism, dysfunctional uterine hemorrhage, pregnancy difficulties, irregular menstrual cycles, alopecia, and acne; as illustrated in Figure 3 (Barnes *et al.*, 1989).

### Necessity to Study PCOS

**Figure 4:**
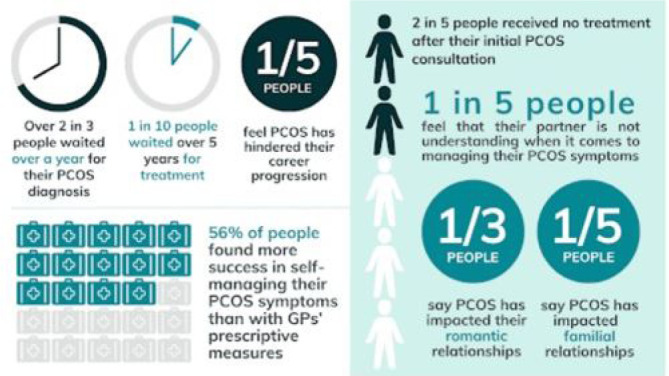
Global burden of PCOS (Knochenhauer et al., 1998).

A PCOS Society survey found that two out of every ten Indian women suffer from PCOS. Six out of ten women with PCOS are adolescent girls. According to studies conducted by the AIIMS Department of Endocrinology and Metabolism, 20-25% of women who are of reproductive age have PCOS; as per shown in Figure 4. Although 35-50% of women with PCOS have fatty livers; 60% of them are obese. Roughly 70% of people are insulin resistant, 60-70% have high testosterone levels, and 40-60% are glucose intolerant. Despite the lack of a defined etiology, some PCOS-affected women have higher-than-normal insulin levels. Excessive insulin production can cause the ovaries to produce higher quantities of androgens, including testosterone. Because insulin resistance might make it more difficult to shed weight, PCOS-afflicted women are more likely to experience obesity (Barnes *et al.*, 1989).

Undiagnosed or untreated, this illness can cause infertility and other long-term health issues. PCOS symptoms in girls and women include weight gain, lethargy, unwanted hair growth, thinning hair, infertility, acne, pelvic discomfort, migraines, sleep issues, and mood swings. In older adults, this condition can lead to diabetes, high blood pressure, and abnormal blood lipid levels. In a study comparing Allopathy, Ayurveda, and Homeopathy, they showed that Allopathy does not cure PCOS but rather manages its symptoms and requires more time and money. Ayurveda and homeopathy, on the other hand, provide a potential remedy with no side effects.

## MEDICINAL PLANTS USED IN THE TREATMENT OF PCOS

Natural products from various sources play a major role in healthcare, mainly in the pharmaceutical, cosmetic, agro, and medical industries. The term “herbs” in commerce refers to any plant, plant component, or extract that is utilized for flavoring, scenting, or for therapeutic reasons. Customary herbal medicines are organic compounds that have been utilized to cure a variety of ailments with little to no industrial processing. Natural products have recently become popular due to their possible therapeutic benefits and low side effects. Phytochemicals produced from medicinal herbs and plants constitute an important route for researching and finding novel pharmacological drugs.

They have been used medicinally for millennia. Many medicinal plants have been shown to have various pharmacological impacts on essential health issues such as antioxidant, antibacterial, anticancer, and antidiabetic characteristics. Because plants act as repositories for diverse phytochemicals, they can be used to cure a variety of ailments. These chemicals include alkaloids, flavonoids, terpenoids, phenolic acids, and other substances. Each of these substances has a distinct chemical makeup and mechanism of action, which may explain their different therapeutic properties. Natural products have recently become popular due to their possible therapeutic benefits and low side effects (Jungbauer & Medjakovic, 2014; Khanage *et al.*, 2019).

There is a growing focus on traditional herbal treatments in international health discussions. The fields of preventive, curative, and rehabilitative medicine are well-established in traditional medicine (Khanage *et al.*, 2019; Knochenhauer *et al.*, 1998). Herbal remedies are a good therapy choice for PCOS due to their gentle nature and lower risk of adverse effects compared to traditional medicine drugs (Khanage *et al.*, 2019). Herbal treatment has reached a tipping point. It is struggling to be recognized as a science--a distinct discipline with its own identity. It has become vital to demonstrate that herbal rehabilitation can compete with other medical professions in terms of scientific rigor and practical application. The advantage of herbal medicine over conventional therapy is that it is safer with fewer side effects, and the presence of numerous active components in medicinal herbs combined gives a potentiating effect (Jungbauer & Medjakovic, 2014). Various medicinal plants and their benefits are illustrated in Table 1.

**Table 1:** List of herbal Plants used in PCOS.

Sr. No.	Common name	Botanical name	Family	Parts used	Phytoconstituents	Chemical Structure	Benefits (Kumari *et al.*, 2024)
1	Liquorice	*Glycyrrhiza glabra*	Leguminosae	Root	glycerin	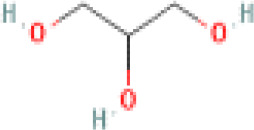	Helps in maintaining the levels of male hormones (Pavithra & Ilango, 2023)
2	Aloe-vera	*Aloe barbadensis Mill.*	Liliaceae	Gel or juice	Polysaccharide compounds, Aloe emodin	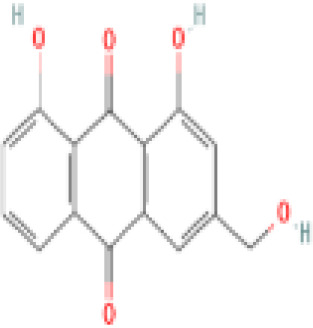	Very hydrating and provides extra lubrication to the body that helps it remove buildup chemicals (Pavithra & Ilango, 2023)
3	Turmeric	*Curcuma longa*	Zingiberaceae	Rhizome	Curcumin	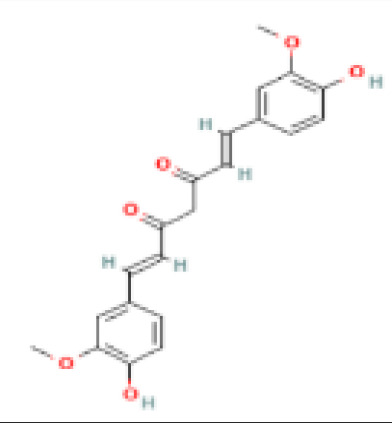	Reduces insulin resistance, lowers blood sugar level and increase HDL or good cholesterol level (Pavithra & Ilango, 2023).
4	Flax seeds	*Linum usitatissimum*	Linaceae	Seed	lignans	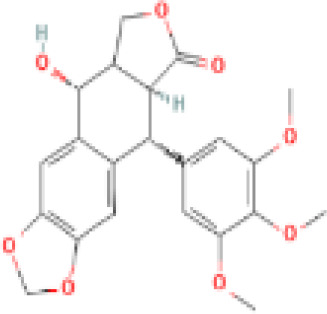	Helps regulate blood sugar, promote weight loss and prevent constipation (Pavithra & Ilango, 2023).
5	Gymnema	*Gymnema sylvestre*	Apocynaceae	leaves	gymnemic acid and quercetin	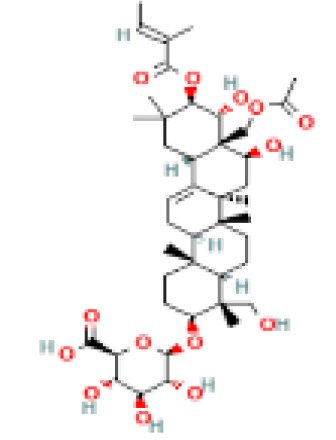	Improves cholesterol and triglyceride levels, reducing heart disease risk (Pavithra & Ilango, 2023).
6	Fennel Seeds	*Foeniculum vulgare*	Apiaceae	Seed	Trans anethole	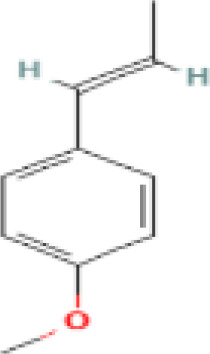	helps in PCOS treatment as they have anti-hirsutism properties and helpsdecrease androgen (male hormones) levels (Bone, 2003).
7	Cinnamon	*Cinnamomum zeylanicum*	Lauraceae	Stem bark	β-caryophyllene	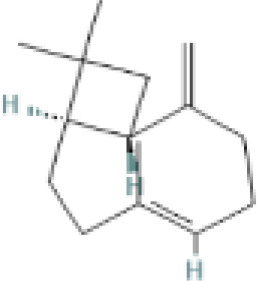	Reduces the cholesterol level, bp and inflammation (Pavithra & Ilango, 2023).
8	Tulsi	*Ocimum sanctum Linn.*	Lamiaceae	Leaves, stem, flower, root, seeds	eugenol	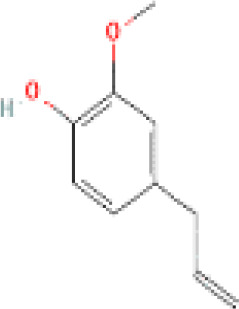	Controls androgens leading to excessive facial hair growth and acne, lower (Wal *et al.*, 2021).
9	Pumpkin Seeds	*Cucurbita maxima*	Cucurbitaceae	Seed	beta-sitosterol	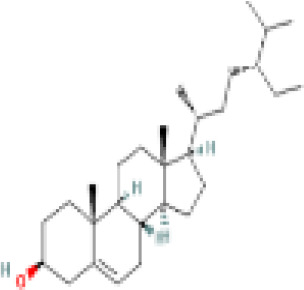	helps reduce excessive cholesterol and insulin levels associated with PCOS (Reddy *et al.*, 2016)
10	Green Tea	*Camellia sinensis*	Theaceae	green leaves	Catechins (L-Epicatechin)	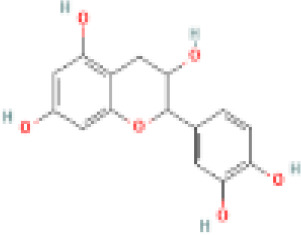	reduces hormone levels associated with ovarian cysts and promotes weight loss (Khanage *et al.*, 2019).
11	Ginseng	*Panax ginseng*	Araliaceae	Leaf, stem	Glutathione and superoxide dismutase	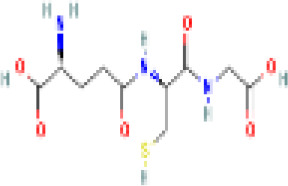	boosts energy, lowers blood sugar level, reduces stress, treats diabetes, manages sexual dysfunction in men (Pavithra & Ilango, 2023).
12	Ashoka tree	*Saraca asoca*	Fabaceae	Seed, bark, flowers	Leucopelargonidin	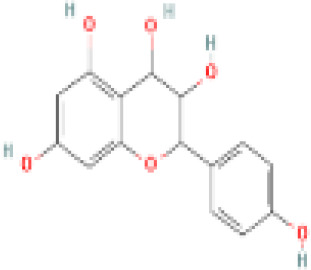	May act against cancer (Pavithra & Ilango, 2023).
13	Drumstick tree	*Moringa oliefera*	Moringaceae	Root, stem, leaves, fruits	Moringyne	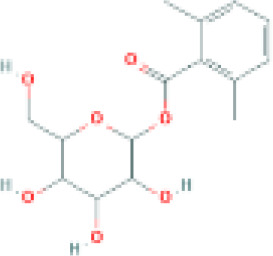	Prevents the growth of colon, lung, stomach cancer cells (Pavithra & Ilango, 2023).
14	Fenugreek	*Trigonella foenum-graceum*	Fabaceae	Seeds, leaves	Trigoneoside IIA	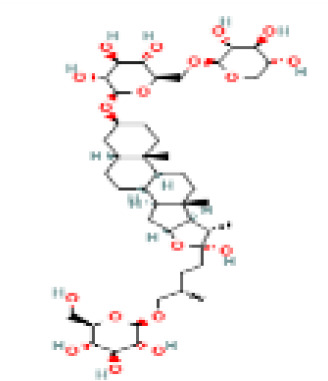	Reduces the risk of heart and blood pressure, pain relief (Pavithra & Ilango, 2023).
15	Sesame seeds	*Sesamum indicum*	Pedaliaceae	Seeds	Sesamin	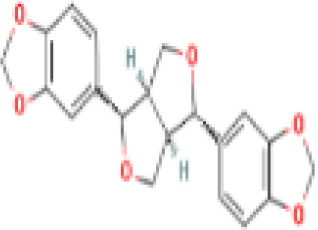	May aid blood sugar control, combats arthritis pain and lower cholesterol (Pavithra & Ilango, 2023).
16	Sugarcane	*Saccharum officinarum*	Poaceae (Gramineae)	Root, Stem	Swertisin	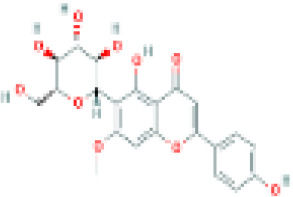	Stores energy in the form of healthy fats (Pavithra & Ilango, 2023).
17	Ginger	*Zingiber officinale*	Zingiberaceae	Rhizome	α-Zingiberene	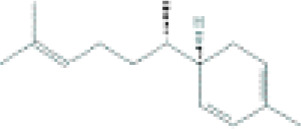	Relieves nausea and vomiting and aids digestion (Pavithra & Ilango, 2023).
18	Neem tree	*Azadirachta indica*	Meliaceae	Fruit, Bark, Leaves, Flow er	Nimbolide	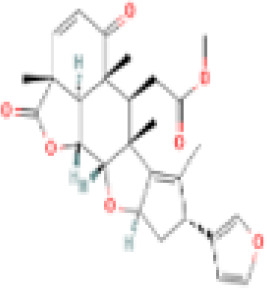	Efficacious against a variety of skin disease, septic sores, infected burns (Pavithra & Ilango, 2023).

Herbs may be used for a longer period with fewer adverse effects, which is important given that PCOS requires long-term therapy. They can be effective in addressing the underlying causes of PCOS, relieving symptoms, and repairing the body by increasing the person’s immune system. We can combine the herbal treatments with a PCOS-friendly diet and fitness regimen to increase the efficiency of the selected botanical therapy.

An Ayurvedic therapy involves using a multifaceted strategy to address:

- Correcting the hormonal imbalance
- Treatment of obesity and avoiding high cholesterol levels
- Treatment to insulin resistance.

### Herbs for PCOS

**Figure 5:**
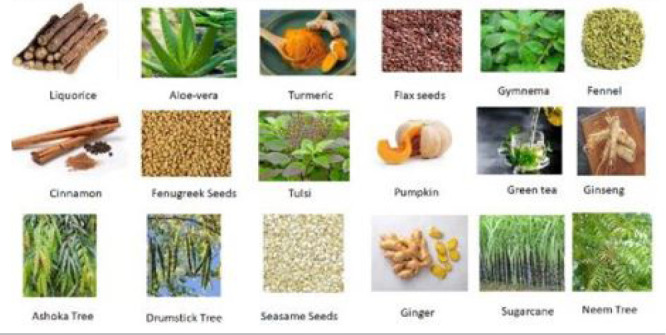
Herbs used in the treatment of PCOS.

The use of herbal plants and natural remedies in the management of Polycystic Ovary Syndrome (PCOS) has gained attention and popularity due to its potential to address some of the symptoms and underlying factors associated with the condition. Following are the herbs that are used extensively in the treatment of PCOS as shown in Figure 5 and some of them are also discussed.

### Liquorice

Liquorice, also known as *Glycyrrhiza glabra L.*, belongs to the Fabaceae family and has been traditionally used for wound healing, pain relief, cough relief, and treating gastritis. Its roots contain medicinal compounds like flavonoids, sterols, gums, starches, and essential oils. Sterols and phytoestrogens in liquorice can help lower triglycerides and cholesterol. The most active ingredient, glycyrrhizin, is 50 times sweeter than sucrose and inhibits the 11beta-hydroxysteroid dehydrogenase type 2 (11βHSD2) enzyme, mimicking mineralocorticoid effects. By inhibiting glucocorticoid metabolism, glycyrrhizin increases glucocorticoid levels in the bloodstream, which in turn boosts insulin secretion and lowers blood sugar (Manouchehri *et al.*, 2023).

The study investigated the effects of liquorice root hydroalcoholic extract on blood sugar, triglycerides, and cholesterol in 50 rats with polycystic ovary syndrome. It included three groups: a normal group, a letrozole group, and two treatment groups receiving liquorice extract after letrozole. The letrozole group had significantly higher blood glucose than the normal group (*p*<0.01), while the treatment groups had lower levels (*p*>0.05). There were no significant differences in triglyceride and cholesterol levels between the groups. The study suggests that liquorice root extract may reduce diabetes-related effects in polycystic ovary syndrome (Barazesh *et al.*, 2016).

### Aloe vera

Aloe vera, also known as *Aloe arborescens*, is a perennial herbaceous plant in the Liliaceae family. This plant contains vitamins A, C, and E. It also has antioxidant properties due to the reduction in lipid peroxidation. Aloe vera is rich in nutrients and minerals, as well as salicylic acid, enzymes, tannins, and polysaccharides. Aloe vera gel’s polysaccharide components have anti-inflammatory and reparative properties. These substances are also antimicrobial and antibacterial (Desai *et al.*, 2012).

The study explored the effects of aloe vera gel on PCOS in rats. Female rats were induced with PCOS by administering letrozole orally for 5 months. Afterward, they were given 1 ml of aloe vera gel daily for 45 days. The study evaluated the rats’ estrous cycle, glucose sensitivity, and steroidogenic activity. The results showed that aloe vera gel, combined with a stimulant, prevented the development of PCOS in the rats. Aloe vera gel protects against PCOS by restoring ovarian steroid balance and modifying steroidogenic activity, attributed to plant compounds like phytosterols and phytophenols. It also inhibits 3β-HSD enzyme activity and regulates estradiol formation (Joshi *et al.*, 2021).

### Turmeric

Curcumin, a naturally occurring polyphenol, has been linked to numerous health benefits. *Curcuma longa* and other Curcuma species’ rhizomes are used to produce it. Curcumin has been shown to have antioxidant, anti-inflammatory, anti-mutagenic, and anticancer properties, and as a result, it has been used as a medicinal herb in Asian countries for centuries. Various clinical and preclinical studies have also confirmed its efficacy in PCOS (Joshi *et al.*, 2021).

Heshmati *et al.* (2020) demonstrated the efficacy of curcumin in PCOS. A randomized double-blind clinical trial was conducted on 72 PCOS patients who were randomly assigned to receive curcumin (500 mg three times daily) or a placebo. Fasting plasma glucose and dehydroepiandrosterone levels decreased by 4.11mg/dL; *p* (adjusted)=0.048 and-26.53microgram/dL; *p* (adjusted)=0.035, respectively, between intervention and control groups. Furthermore, the authors reported that there were no serious adverse effects during the entire 12-week treatment period.

### Flax seeds

Flax, scientifically known as *Limum usitatissimum*, is part of the Linaceae family. Flaxseed contains high levels of fat, protein, and fiber. Flaxseed typically contains 30-40% fat, 20-25% protein, 20-28% fiber, 4-8% moisture, and 3-4% ash, along with vitamins A, B, D, E, minerals, and amino acids. Flaxseed oil is high in linolenic acid, omega-3 fatty acids, lignans, and mucilage.

Studies show that polycystic ovaries increase androgen levels, leading to hirsutism, menstrual disorders, and obesity. A study examined the effects of taking 30 grams of flaxseed daily on hormone levels in a 31-year-old woman with PCOS. Over four months, the patient consumed 83% of the prescribed flaxseed dose. Measurements of height, weight, and fasting blood samples were taken before and after the follow-up period. Results showed a significant reduction in body mass index, insulin, serum testosterone, and free serum testosterone. The patient also reported decreased hirsutism, which correlated with the reduction in androgen levels (Emam *et al.*, 2021).

### Gymnema

*Gymnema sylvestre* is a herbal remedy used in Ayurvedic medicine to manage diabetes. Antidiabetic herbs may be more effective in treating polycystic ovarian syndrome (PCOS) because they promote obesity and reduce insulin resistance. According to a study, YAP-1, a newly discovered pathogenic component of PCOS, is downregulated in G. sylvestre, suggesting that it may be a viable plant to prevent PCOS. In summary, these results show that the key pathogenic gene, YAP1, is mediated by G. sylvestre treatment to improve the mitochondrial architecture in the PCOS ovary. Furthermore, the research offers fresh perspectives on enhanced mitochondrial architecture triggered by G. sylvestre’s therapeutic intervention for PCOS management (Jangam *et al.*, 2024).

### Fennel Seeds

Fennel is a herbaceous plant belonging to the Apiaceae family, scientifically known as *Foeniculum vulgare*. In traditional medicine, fennel is used to treat various digestive, endocrine, reproductive, and menstrual disorders because it is an estrogenic compound. The antimicrobial, antifungal, antioxidant, antithrombotic, anti-diabetic, and anti-tumor properties of fennel and its compounds.

The effect of metformin and fennel on uterine tissue and serum concentrations of estrogen and progesterone was investigated in rats with PCOS. According to the findings, fennel improved progesterone and uterine endometrial thickness while decreasing estrogen and uterine epithelial thickness in PCOS rats. Fennel may therefore protect the uterine tissue of PCOS-affected rats (Meena *et al.*, 2019).

### Cinnamon

*Cinnamomum zeylanicum*, commonly known as cinnamon, is a member of the Lauraceae family of plants and is one of the most significant and ancient herbal medicines utilized in traditional medicine. Its volatile oil, which contains components such as cinnamaldehyde, eugenol, and safrole, has many medicinal properties, and the plant’s skin is one of its many parts. In one study, for eight weeks, 15 PCOS-afflicted women took 333 mg of oral capsules containing cinnamon extract three times a day. The patients underwent insulin sensitivity testing both before and after receiving cinnamon extract. Two hours after ingesting cinnamon extract, the patients’ insulin levels significantly decreased, according to the results. Due to an increase in phosphatidylinvestyl 4-kinase activity, cinnamon reduced insulin levels in patients (Wang *et al.*, 2007).

### Tulsi

Tulsi is a sacred herbal plant with a variety of medicinal applications, the two main ones being the treatment of obesity and hypoglycemia. Because of its anti-androgenic qualities, it is used to treat polycystic ovarian syndrome. It controls obesity and reduces androgen synthesis. The body does not use the androgens because the correct ovulation process does not occur. The underused androgens are the cause of acne and hirsutism. The appropriate regulation and utilization of androgen levels is Tulsi’s function. Additionally, it has antioxidant properties (Wal *et al.*, 2021).

### Ginseng

Ginseng, scientifically known as *Panax ginseng*, is a medicinal plant in the Araliaceae family. It is both fragrant and long-lasting. This plant is rich in antioxidants and boosts resistance. This formulation effectively reduces plasma LH levels and improves endocrine status for treating ovulation disorders in PCOS patients (Andhalkar *et al.*, 2021).

Ginseng was tested on Sprague Dawley rats with PCOS induced by an intramuscular injection of estradiol. The animals were studied for nervous growth factor (NGF) and ovarian morphology. In animals with PCOS, NGF increased in the ovaries and brain, whereas ginseng decreased NGF (Pak *et al.*, 2005).

### Ashoka tree

The Ashoka tree is a member of the Leguminous plant family. The dried bark of the tree contains calcium-based compounds, tannins, and catechol. The Asoka tree contains several essential ions, such as magnesium, calcium, sodium, and phosphate.

Ashoka bark is primarily used to treat PCOS, irregular menstrual disorders, excessive bleeding, uterine spasms, mild to moderate pain, and dysmenorrhea. Asoka is regarded as one of the most effective uterine tonics because it aids in the treatment of irregular menstrual cycles and miscarriages. It acts as an astringent to reduce excessive menstrual bleeding. It also causes contractions of the uterine muscles. The studies also report affecting uterine fibroid and menorrhagia in cases of PCOS (Jalilian *et al.*, 2013; Khanage *et al.*, 2019; Mitsi & Efthimiou, 2014; Satapathy *et al.*, 2017; Wal *et al.*, 2021).

## FORMULATIONS AVAILABLE

Various formulations are available in the market like capsules, syrups, tablets, emulsions etc. for the treatment of PCOS. The list of formulations available are mentioned in Table 2 (Andhalkar *et al.*, 2021; Armanini *et al.*, 2004; Dunne & Slater, 2006; Goldzieher & Green, 1962; Kafali *et al.*, 2004; Knochenhauer *et al.*, 1998; Kumar *et al.*, 2019; Maharjan *et al.*, 2010; Pandya *et al.*, 2015; Rizzo *et al.*, 2008; Saini *et al.*, 2016; Salem *et al.*, 2019; Tiwari *et al.*, 2023).

**Table 2:** List of formulations available.

Sr. No.	Formulations available	Images	Uses
1	Nano emulsion vaginal suppositories of progesterone	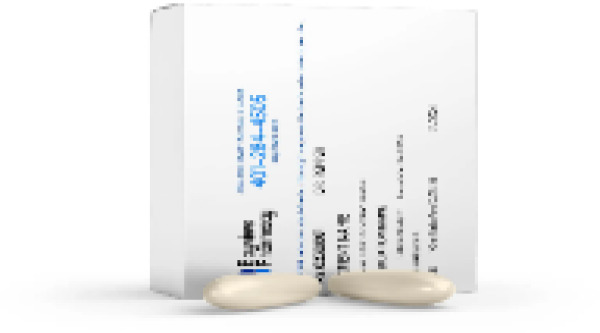	It is used to promote fertility and it has also been prescribedforinductionamenorrhea,regularbleeding,and sub atrophy or full secretory changes of the endometrium (Kumar *et al.*, 2019).
2	Ovaryl tablet an herbal formulation	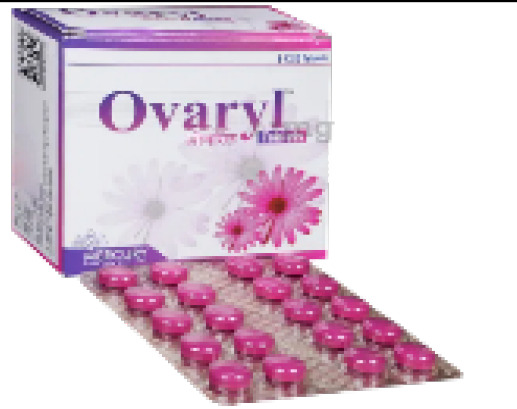	PCOS symptoms like weight, hirsutism, irregularity of periods and ovulation symptoms were taken care off and decreases in weight, no cyst formation, Decreases Serum Insulin levels, Decreases right and left ovary volume (Pandya *et al.*, 2015).
3	Aloe vera gel (AVG) formulation	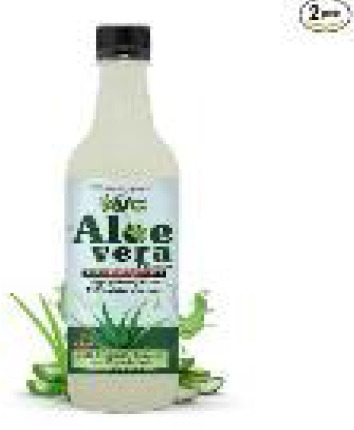	Exerts a protective effect against the PCOS phenotype by restoring the ovarian steroid status and altering key steroidogenic activity.
4	Phytoestrogen-rich supplements	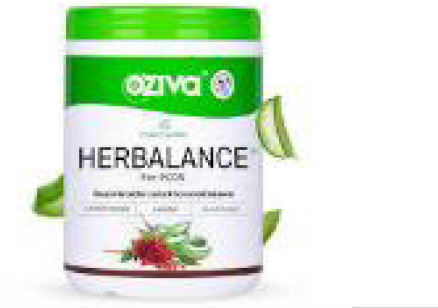	It provides health benefits such as menstrual regulation, reducing weight, normalizing the blood glucose level, and reduces oxidative stress.
5	Capsules	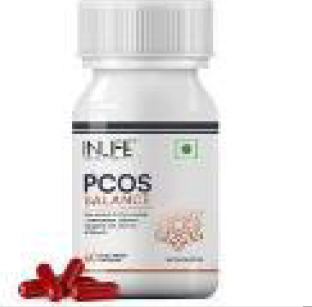	Balances hormones, balances androgen level and reduces facial hair growth, prevents hair fall, treats PCOS
6	Syrups	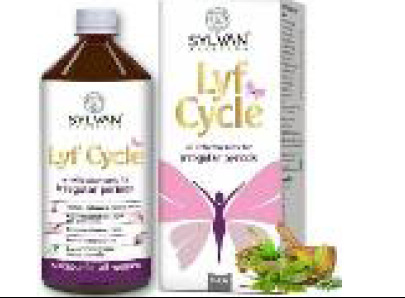	Anti-androgenic activity, hypoglycemic, hypolipidemic, and insulin resistance improving the property and anxiolytic/anti-stress properties.
7	Metformin tablets	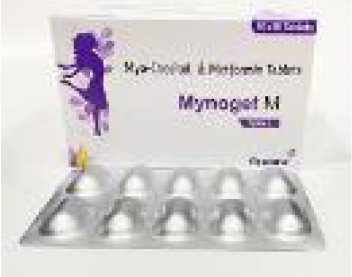	It helps support regular ovulation and menstrual cycles.
8	Clomiphene citrate tablets	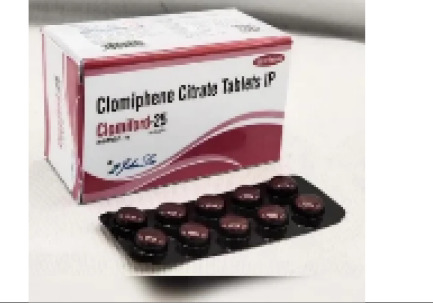	Induces ovulation (egg production) in women who do not produce ova (eggs) but wishes to become pregnant (infertility).
9	Oral contraceptives	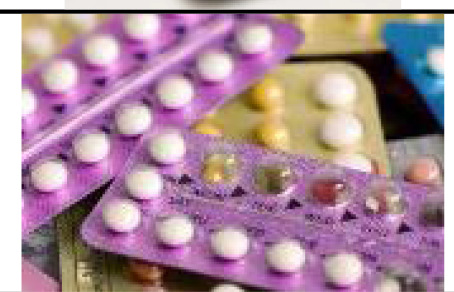	The hormones in birth control pills may help regulate menstrual bleeding. The pills also may help reduce excessive hair growth and acne.
10	Dexamethasone matrix tablets (Pak *et al.*, 2005)	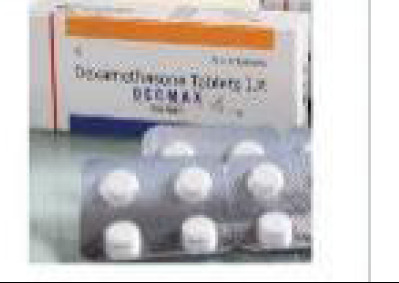	Improves folliculogenesis, ovulation, and pregnancy rate in CC resistant PCOS.
11	Inositol powder	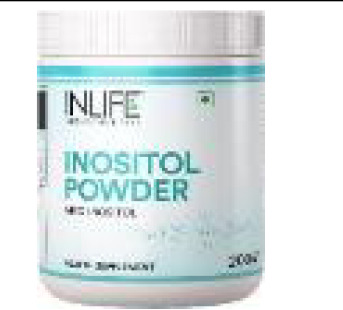	Improves blood sugar, reduces blood pressure and lowers triglyceride levels. It may also promote ovulation and increase pregnancy rates.

## EXPERIMENTAL FORMULATIONS FOR PCOS

There are some formulations that are developed by the researchers in their study; and experimental formulations are not available in the market. The list of experimental formulations is given in Table 3.

**Table 3:** List of experimental formulations.

Sr. No.	Formulation	Uses
1	Intravaginal administration of metformin hydrochloride-loaded cationic niosomes amalgamated with thermosensitive gel	It offers analogous advantages as compared to oral metformin hydrochloride (MTF-HCl) solution in the treatment of polycystic ovary syndrome (PCOS) at a lower dosage regimen with probably negligible side-effect (Nowak *et al.*, 2007).
2	Progesterone-loaded nanovesicle transethosomes (NVTE_S_)	PRG-loaded NVTEs vaginal gel might be a promising formulation for luteal phase support and increased pregnancy rate in anovulatory PCOS (Wang *et al.*, 2007).
3	Herbal Extracts Loaded Phyto-phospholipid complexes	Herbal preparations with low oral bioavailability have a fast first-pass metabolism in the gut and liver. To offset these effects, a method to improve absorption and, as a result, bioavailability must be devised, with antioxidant effects, balances hormones, and anti-inflammatory properties (Choi *et al.*, 2020).
4	Curcumin Encapsulated Self-Assembled Nanoparticles	decreasing serum LH, prolactin, testosterone, and insulin levels (Raja *et al.*, 2021).

## CONCLUSION

Polycystic ovarian syndrome (PCOS) is the leading cause of female infertility. Herbal medicines are promising alternatives for managing PCOS but they require more scientific validation. Herbal medicines are associated with very few side effects, and they are a safe treatment. There are so many synthetic medicines also available in the market but with some side effects. This review emphasizes how herbal remedies can provide treatment for women with PCOS with comprehensive, patient-centered therapy. Various symptoms associated with PCOS can be overcome by herbal medicinal plants. Any researcher who wants to work in this area can refer to this review for further development.
